# EchoSAM2: enhancing anatomical consistency in few-shot cardiac ultrasound segmentation via sequence-wise memory and registration

**DOI:** 10.3389/fmed.2026.1868533

**Published:** 2026-09-03

**Authors:** Chunmin Li, Ziyu Zhou, Lijuan Guo, Qiulian Wang, Xiaoji Bai, Wenhao Wang

**Affiliations:** 1 Department of Ultrasound, Shanxi Children's Hospital (Shanxi Women and Children's Hospital), Taiyuan, China; 2 School of Electronic Information and Electrical Engineering, Shanghai Jiao Tong University, Shanghai, China; 3 Department of Obstetrics and Gynecology, Second Hospital of Shanxi Medical University, Taiyuan, China

**Keywords:** echocardiography, few-shot video segmentation, medical image registration, segment anything 2, temporal memory propagation

## Abstract

**Introduction:**

Few-shot segmentation in echocardiography is highly valuable for rapid clinical assessment, yet remains challenging because expert annotations are scarce, cardiac morphology varies across patients, and ultrasound videos exhibit complex temporal motion. Although the Segment Anything family provides strong generic priors, its direct deployment on echocardiographic videos often suffers from unstable frame-wise predictions and limited adaptation under low-label conditions.

**Methods:**

We propose EchoSAM2, a few-shot framework tailored to cardiac ultrasound video analysis. The method introduces support-conditioned, sequence-level memory propagation to preserve anatomical coherence throughout the cardiac cycle. To stabilize inference, we design an Adaptive Start-Frame Selection (ASFS) module that automatically identifies a favorable initial frame and incorporate a lightweight registration network to efficiently align support and query frames.

**Results:**

Experiments on the CAMUS and EchoNet-Dynamic datasets demonstrate that EchoSAM2 achieves new state-of-the-art performance for few-shot segmentation and clearly outperforms existing video segmentation and SAM-adaptation baselines under low-annotation settings. EchoSAM2 also narrows the performance gap between few-shot and fully supervised models.

**Discussion:**

These results demonstrate the effectiveness of support-conditioned temporal propagation, adaptive start-frame selection, and efficient frame alignment for few-shot echocardiographic video segmentation. EchoSAM2 shows practical potential for automated cardiac quantification and computer-aided diagnosis in data-constrained clinical settings.

## 1 Introduction

Echocardiography is a frontline modality for cardiac assessment because it is noninvasive, inexpensive, and capable of real-time imaging. Reliable delineation of cardiac structures (e.g., ventricles and myocardium) is fundamental for quantitative analysis of heart function, including function indices used in daily diagnosis and follow-up. However, dense pixel-wise annotation in ultrasound is time-consuming and depends on experienced clinicians, which limits the scalability of fully supervised pipelines in routine clinical environments.

Few-shot segmentation attempts to overcome this bottleneck by learning transferable segmentation behavior from very limited labeled examples. Despite encouraging progress, echocardiographic scenarios remain challenging for three main reasons: (i) ultrasound data are often degraded by speckle noise, low contrast, and substantial inter-patient appearance variation; (ii) many few-shot methods process frames independently and do not utilize temporal continuity in video data; and (iii) foundation models such as SAM are not optimized for ultrasound dynamics, so direct adaptation is constrained by domain shift and insufficient temporal reasoning.

Existing approaches still exhibit notable limitations in this setting. Classical prototype-based few-shot models usually summarize support information into compact vectors (1–4); this design is efficient but often loses spatial details that are critical for delineating thin or weak-contrast myocardial boundaries. Video-based methods improve temporal smoothness (5–9), yet many depend on high-quality initial masks or dense supervision, so errors in early frames can be propagated and amplified over time. SAM-adaptation methods benefit from large-scale pretraining (10–14), but in echocardiography they may generate coarse masks and unstable frame-to-frame predictions because natural-image priors do not fully capture ultrasound texture statistics and periodic cardiac motion. In addition, most pipelines assume fixed or heuristic start frames and do not explicitly optimize initialization quality, which further increases sensitivity in sequential inference.

These observations motivate a framework that jointly addresses *support-query mismatch, temporal error accumulation*, and *initialization sensitivity*. To this end, we present **EchoSAM2**, a sequence-aware few-shot framework that combines support-conditioned memory with temporal propagation. Instead of predicting each frame in isolation, EchoSAM2 propagates support and query cues along the sequence, thereby improving temporal coherence and structural stability around challenging boundaries. The framework further includes two practical components: (1) an **Adaptive Start-Frame Selection (ASFS)** module that chooses a reliable initialization frame for bidirectional inference to reduce early-error amplification, and (2) a lightweight **registration network** that aligns support and query frames before memory construction to improve support-query correspondence at low computational cost. Comprehensive experiments on CAMUS and EchoNet-Dynamic confirm that EchoSAM2 achieves leading performance under highly limited supervision. The model consistently surpasses existing few-shot, video-segmentation, and SAM-based baselines while narrowing the performance gap with fully supervised alternatives.

Our main contributions are:

- We introduce EchoSAM2, a sequence-wise few-shot framework for echocardiography that leverages support-conditioned memory propagation.
- We develop ASFS for adaptive inference initialization, improving robustness and final segmentation accuracy.
- We integrate an efficient registration network to enhance support-query alignment while preserving runtime efficiency.
- We report state-of-the-art few-shot results on two public benchmarks under extreme annotation scarcity.

## 2 Related works

### 2.1 Few-shot medical image segmentation

Medical image segmentation under sparse labels has attracted increasing attention due to annotation cost, availability, and privacy constraints. Within this landscape, Few-Shot Medical Image Segmentation (FSMIS) has become a practical direction, aiming to segment unseen anatomies from only a few labeled support examples. Existing FSMIS approaches are commonly categorized into prototype-based methods and support-query interaction methods, both of which improve data efficiency from different perspectives.

Prototype-based methods build class representations from support images and match them to query features. PANet (1) established this paradigm in few-shot segmentation. Subsequent medical adaptations introduced anatomy-aware and spatially constrained variants. For instance, Ouyang et al. (2) proposed local anatomical prototypes, Yu et al. (3)cues, and Hansen et al. (15) explored foreground-focused prototypes with 3D supervoxel priors. Huang et al. (4) later adopted vector quantization to obtain compact prototype dictionaries with improved generalization. In parallel, interaction-based methods strengthen support-query communication through explicit feature exchange. Roy et al. (16) employed multi-level squeeze-and-excitation conditioning, while later studies integrated cross-image attention (17), graph reasoning (18), and cross-convolution fusion (19).

Even with these advances, most FSMIS models still process slices/frames as independent samples, overlooking strong continuity in sequential data such as video and volumetric scans. In our view, earlier predictions in a sequence provide useful priors for subsequent frames and should be explicitly utilized, particularly in cardiac ultrasound where adjacent frames are anatomically correlated.

### 2.2 Few-shot video object segmentation

Video Object Segmentation (VOS) (20–22) addresses object tracking and mask propagation from an annotated initial frame. Early methods such as OSMN (23) and LML (24) relied on prompt-derived prototypes for frame-wise matching. Memory-based VOS methods then became dominant: STM (5) introduced explicit memory, STCN (6) improved memory efficiency, XMem (7) enhanced multi-granularity memory design, and SAM2 (25) further boosted capability as a large video segmentation model built upon SAM (26).

To reduce dense annotation requirements, Few-shot VOS (FSVOS) (8, 9) studies category-generalized segmentation with only a small support set. Existing FSVOS methods mainly follow prototype-centric (8, 9, 27) or affinity/matching-centric (9, 28) designs. DANet (8) first formalized the task; TTI (28) and VIPMT (9) improved temporal coherence with multi-scale prototypes; HPAN (27) and CoCoNet (9) further explored graph attention and optimal transport. These studies provide strong methodological references for memory design and temporal modeling.

However, transferring these methods directly to medical video remains suboptimal (29) because ultrasound videos differ from natural scenes in appearance statistics, background complexity, and motion regularity. Cardiac ultrasound often exhibits structured and repetitive motion patterns across patients, suggesting that domain-specific sequence modeling can be more effective than generic natural-video priors and can better exploit anatomical continuity.

### 2.3 Medical image registration

Image registration estimates spatial correspondence by transforming one image to align with another. This is especially relevant for 2D cardiac ultrasound, where alignment quality directly affects downstream segmentation and quantification. Compared with CT/MRI, ultrasound registration is harder because of strong speckle noise, artifacts, and large appearance variability.

In few-shot ultrasound settings, robust support-query alignment is important for transferring anatomical information. Existing deep registration methods are generally supervised, unsupervised, or cascaded. Supervised approaches regress transformation parameters from labeled pairs (e.g., (30)), and deformable variants may predict dense displacement fields inspired by optical flow (31). Since ground-truth deformation fields are difficult to obtain, unsupervised methods are widely used. VoxelMorph (32) is a representative baseline, followed by models integrating self-attention (33), cycle consistency (34), and multi-modal modeling (35). Architecturally, cascaded designs improve accuracy by stacking sub-networks, at the expense of additional complexity and latency; examples include RCNet (36), C2FViT (37), and hybrid affine-deformable pipelines (38, 39).

For 2D ultrasound few-shot segmentation, lightweight yet effective registration integration remains underexplored. In this study, we incorporate a representative deformable registration strategy into our segmentation framework as a supporting component for better support-query alignment rather than a standalone objective.

## 3 Methods

Given a query ultrasound video $Q\in {\mathbb{R}}^{N\times H\times W}$ and, for each target structure *c*, a few-shot support set $S^{\left(c\right)}={\left\{\left(I_{s}^{i},Y_{s}^{i,\left(c\right)}\right)\right\}}_{i=1}^{K}$, where $I_{s}\in {\mathbb{R}}^{H\times W}$ and $Y_{s}^{\left(c\right)}\in {\left\{0,1\right\}}^{H\times W}$ is the binary mask of structure *c*. Our objective is to segment the target structures across all query frames, producing for each structure *c* a binary mask sequence ${\bar{Y}}^{\left(c\right)}={\left\{{Ȳ}_{q}^{j,\left(c\right)}\right\}}_{j=1}^{N}$ with ${Ȳ}_{q}^{j,\left(c\right)}\in {\left[0,1\right]}^{H\times W}$.

A straightforward strategy is independent frame-wise few-shot inference, but this ignores temporal continuity and often yields inconsistent masks around thin structures and low-contrast boundaries. In addition, support images and query videos may come from different subjects or acquisition sessions, creating notable appearance and spatial discrepancies that can degrade support-query matching quality.

**Figure 1:**
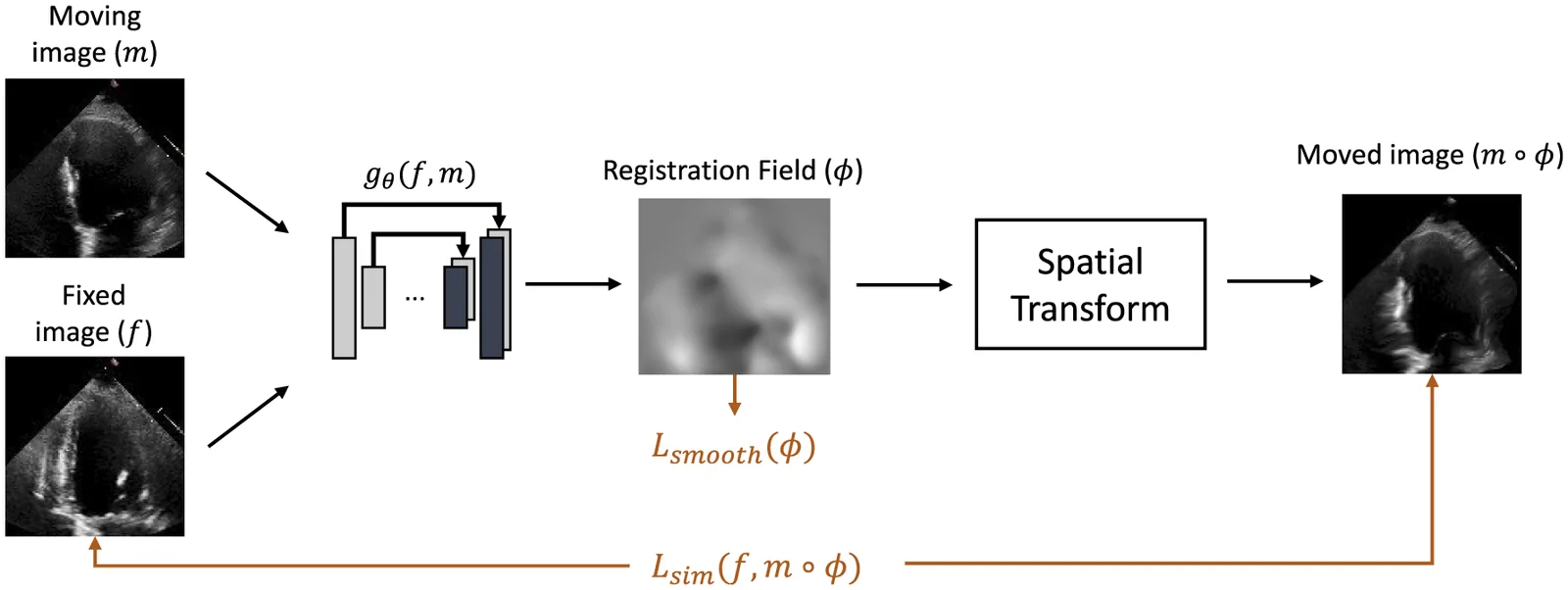
Stage I: Self-supervised ultrasound registration. The network estimates deformation field ϕ to align moving image *m* to fixed image *f*, optimized with image similarity and deformation smoothness losses.

**Figure 2:**
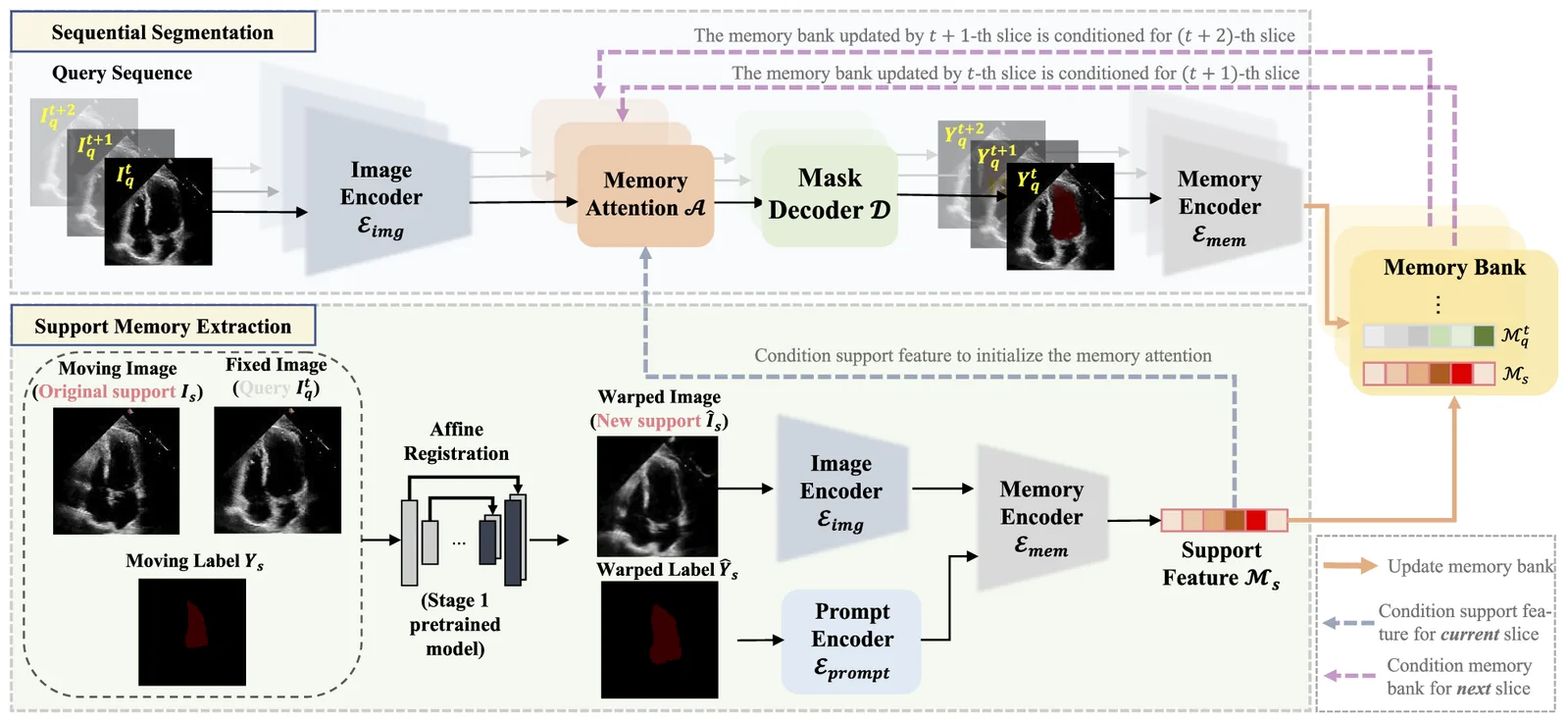
EchoSAM2 overview. Support memory is created after registration and encoding. Bidirectional sequence inference then uses a memory bank containing support and query memories for temporally consistent segmentation.

To address both issues, EchoSAM2 adopts two stages: (i) self-supervised ultrasound registration pretraining on unlabeled data (Figure 1), and (ii) sequence-wise SAM2 segmentation with support-query alignment and memory propagation (Figure 2). Stage I learns reusable geometric priors from abundant unlabeled data, while Stage II uses these priors to build better support memories and performs temporally coherent decoding over the full sequence.

### 3.1 Stage I: self-supervised ultrasound registration

Support and query data are usually not perfectly aligned in anatomy location or appearance. We therefore train a registration model to estimate spatial transformations between ultrasound images and reduce this gap as shown in Figure 1. This module is trained once and reused during inference, so it introduces only lightweight overhead at deployment.

Using unlabeled slices, we sample image pairs at the same relative frame position from different sequences. For each pair, one image is moving (*m*) and the other fixed (*f*). Sampling by relative phase helps avoid unrealistic deformation targets and stabilizes training. The network *g*_θ_ predicts a dense deformation field ϕ:


$$\begin{array}{l} \phi =g_{\theta }\left(f,m\right) \end{array}$$ (1)

The warped image is *m*°ϕ, where ° denotes differentiable spatial transformation. Training uses a self-supervised objective with similarity and smoothness terms:


$$\begin{array}{l} L_{reg}=L_{sim}\left(f,m{}^{\circ}\phi \right)+\lambda L_{smooth}\left(\phi \right) \end{array}$$ (2)

The similarity term is $L_{sim}\left(f,m{}^{\circ}\phi \right)=||f-m{}^{\circ}\phi {||}_{2}^{2}$, and smoothness regularization is $L_{smooth}\left(\phi \right)=\underset{p}{\sum }||\nabla \phi \left(p\right){||}^{2}$, with *p* indexing spatial coordinates. The first term encourages anatomical correspondence in intensity space, while the second suppresses unrealistic local folding and discontinuous displacement.

After training, the model aligns support frames to query frames and provides improved initialization for subsequent segmentation. During Stage II, the same transformation is applied to both support image and mask, so the mask prompt remains geometrically consistent with aligned support features.

### 3.2 Stage II: sequence-wise few-shot segmentation

With the registration module fixed, we perform sequence-level segmentation on ultrasound videos using SAM2. As shown in Figure 2, this stage includes support memory extraction and sequential segmentation. In practice, support memory is generated once per support-query pair and reused throughout temporal inference, which keeps the pipeline efficient.

#### 3.2.1 Support memory extraction

Given support image-mask pair (*I*_*s*_, *Y*_*s*_) and query frame $I_{q}^{j}$, we first warp support data via the learned transformation:


$$\begin{array}{l} \left({Î}_{s},{Ŷ}_{s}\right)=\left(T\left(I_{s}\right),T\left(Y_{s}\right)\right) \end{array}$$ (3)

where *T* is induced by the predicted deformation. For multi-shot settings, this process is repeated for each support pair, and resulting memories can be aggregated by averaging or concatenation before attention.

Aligned support image features are extracted as


$$\begin{array}{l} F_{s}=E_{img}\left({Î}_{s}\right) \end{array}$$ (4)

and aligned support mask embeddings are obtained as


$$\begin{array}{l} F_{prompt}=E_{prompt}\left({Ŷ}_{s}\right). \end{array}$$ (5)

The memory encoder fuses both cues:


$$\begin{array}{l} M_{s}=E_{mem}\left(F_{s},F_{prompt}\right) \end{array}$$ (6)

Unlike sparse prototype vectors, $M_{s}$ preserves dense spatial semantics and offers richer structural guidance. This is particularly useful for ultrasound, where fine boundary cues may be weak but still recoverable from local context when image and prompt features are jointly encoded.

#### 3.2.2 Sequential segmentation

Instead of isolated frame prediction, EchoSAM2 decodes the entire clip sequentially. Current inference depends on support memory and historical query memories. Formally, the state at time *j* can be written as $H^{j}=\left\{M_{s},B^{j-1}\right\}$, where $B^{j-1}$ denotes the query-memory queue before processing frame *j*:

1. **Initialization**. The ASFS module (Section 3.3) selects the starting frame. Inference then runs in both temporal directions so each frame is decoded with nearby temporal context.
2. **Query Encoding**. For frame $I_{q}^{j}$, features are
  $$\begin{array}{l} F_{q}^{j}=E_{img}\left(I_{q}^{j}\right) \end{array}$$ (7)
3. **Memory Attention**. Condition query features with support and, when available, previous query memories:
  $${\tilde{F}}_{q}^{j}=\{\begin{array}{ll} A(F_{q}^{j}|{ℳ}_{s}), & \text{if }I_{q}^{j}\text{ is the starting frame}\\ A(F_{q}^{j}|{ℳ}_{s},\{{ℳ}_{q}^{j-1},{ℳ}_{q}^{j-2},...\}), & \text{otherwise} \end{array}$$ (8)
4. **Mask Decoding**. The decoder predicts
  $$\begin{array}{l} {Ȳ}_{q}^{j}=D\left({\tilde{F}}_{q}^{j},{\text{T}}_{mask}\right) \end{array}$$ (9)
5. **Memory Update**. Build query memory and push it into the bank:
  $$\begin{array}{l} M_{q}^{j}=E_{mem}\left(F_{q}^{j},E_{prompt}\left({Ȳ}_{q}^{j}\right)\right) \end{array}$$ (10)
  The bank contains persistent support memory $M_{s}$ and a FIFO queue of recent query memories with length *L*. If the queue is full, the oldest entry is removed. This strategy balances long-range context and computational cost, preventing uncontrolled memory growth while preserving short-term temporal dynamics.

This sequence-wise design enforces temporal consistency and better handles gradual anatomical changes. Relative to independent frame decoding, it reduces flickering predictions and improves boundary continuity across the cardiac cycle. Following the object-centric design of SAM2, EchoSAM2 segments one structure at a time: the binary support mask of structure *c* is propagated along the query video to yield the binary prediction sequence ${\bar{Y}}^{\left(c\right)}$. When multiple structures are annotated, the same procedure is applied independently to each, and the resulting binary maps are merged into a single multi-structure labeling, with any per-pixel conflicts resolved by the higher predicted confidence. This decomposes a *C*-way segmentation task into *C* SAM2-compatible binary sub-tasks.

### 3.3 Adaptive start-frame selection

Sequential inference is sensitive to the first predicted frame: a poor start can contaminate memory and harm later outputs. To improve initialization, we introduce ASFS, a data-driven strategy for choosing the most reliable initial frame before bidirectional decoding.

For each query frame $I_{q}^{j}$, we register the support image and compute ROI-restricted MSE:


$$\begin{array}{l} d_{\text{MSE}}^{j}=\frac{1}{\left|\Omega_{j}\right|}\underset{\left(x,y\right)\in \Omega_{j}}{\sum }{\left[{Î}_{s}^{q_{j}}\left(x,y\right)-I_{q}^{j}\left(x,y\right)\right]}^{2} \end{array}$$ (11)

where Ω_*j*_ denotes the ROI. In implementation, Ω_*j*_ is defined by the transformed support-mask bounding region (with a small margin) to reduce background interference.

The start index is selected by


$$\begin{array}{l} j^{*}=\arg\underset{j}{\min}d_{\text{MSE}}^{j} \end{array}$$ (12)

and bidirectional decoding proceeds from j^*^. Intuitively, smaller appearance discrepancy between aligned support and query frames often leads to higher-quality initial masks, which in turn improves memory quality for subsequent frames. Therefore, ASFS reduces cumulative error and increases inference stability over long sequences.

## 4 Experimental setups

### 4.1 Datasets and evaluation metrics

#### 4.1.1 Datasets

We evaluate on two public echocardiography benchmarks: CAMUS (40) and EchoNet-Dynamic (41).

**CAMUS Dataset** CAMUS contains 500 studies with apical two-chamber (A2C) and apical four-chamber (A4C) videos. Each sequence has 17–25 frames and provides three-class annotations (LA, LV endocardium, MYO), corresponding to a 3-way few-shot segmentation task. Following the setting of prior echocardiography work (42), this study adopts the CAMUS-Semi configuration, in which only the end-diastolic (ED) and end-systolic (ES) frames are used for evaluation. Following prior practice, data are split into train/val/test with a 7:1:2 ratio. In addition to this CAMUS-Semi benchmark, CAMUS also provides dense per-frame annotations across the cardiac cycle; we use these continuous annotations exclusively for the temporal evaluation in Section 5.3, where segmentation is assessed on consecutive frames rather than on ED/ES frames alone.

**EchoNet-Dynamic Dataset** EchoNet-Dynamic includes 10,030 A4C videos. LV annotations are available at end-diastole (ED) and end-systole (ES) only. Video length ranges from 60 to 200 frames. We use the official split and evaluate on annotated ED/ES frames.

Training is performed in two stages: registration pretraining on unlabeled training frames (Stage I), followed by few-shot segmentation evaluation on test videos (Stage II). In stage II, the labeled support frames are sampled exclusively from the training split, while all query videos for evaluation are drawn from the test split, ensuring no patient overlap between support and query across all experiments.

#### 4.1.2 Evaluation metrics

We report four standard segmentation metrics: mean Dice Similarity Coefficient (DSC), mean Intersection-over-Union (mIoU), 95th percentile Hausdorff Distance (HD95), and Average Symmetric Surface Distance (ASSD).

**Mean Dice Similarity Coefficient (DSC)**


$$\begin{array}{l} \mathrm{DSC}\left(X,Y\right)=\frac{2|X\cap Y}{\left|X|+|Y\right|} \end{array}$$ (13)

where *X* and *Y* are predicted and ground-truth masks. Larger values indicate better overlap.

**Mean Intersection-over-Union (mIoU)**


$$\begin{array}{l} \mathrm{IoU}\left(X,Y\right)=\frac{\left|X\cap Y\right|}{\left|X\cup Y\right|} \end{array}$$ (14)

mIoU is the average IoU over all samples.

**95th Percentile Hausdorff Distance (HD95)** Let *S*_*X*_ and *S*_*Y*_ denote boundary point sets of prediction and ground truth. Directed Hausdorff distance is


$$\begin{array}{l} d\left(S_{X},S_{Y}\right)=\underset{x\in S_{X}}{\max}\underset{y\in S_{Y}}{\min}||x-y|| \end{array}$$ (15)

and HD95 is the 95th percentile of symmetric surface distances.

**Average Symmetric Surface Distance (ASSD)**


$$\begin{array}{l} \;\mathrm{ASSD}\left(S_{X},S_{Y}\right)=\\ \frac{1}{\left|S_{X}|+|S_{Y}\right|}\left(\underset{x\in S_{X}}{\sum }\underset{y\in S_{Y}}{\min}||x-y||+\underset{y\in S_{Y}}{\sum }\underset{x\in S_{X}}{\min}||y-x||\right) \end{array}$$ (16)

Lower HD95/ASSD indicates better boundary alignment.

### 4.2 Implementation details

To ensure fair comparison, we follow preprocessing and evaluation settings from prior echocardiography work (42) on CAMUS and EchoNet-Dynamic. Frames are resized to 256 × 256 and normalized.

**Figure 3:**
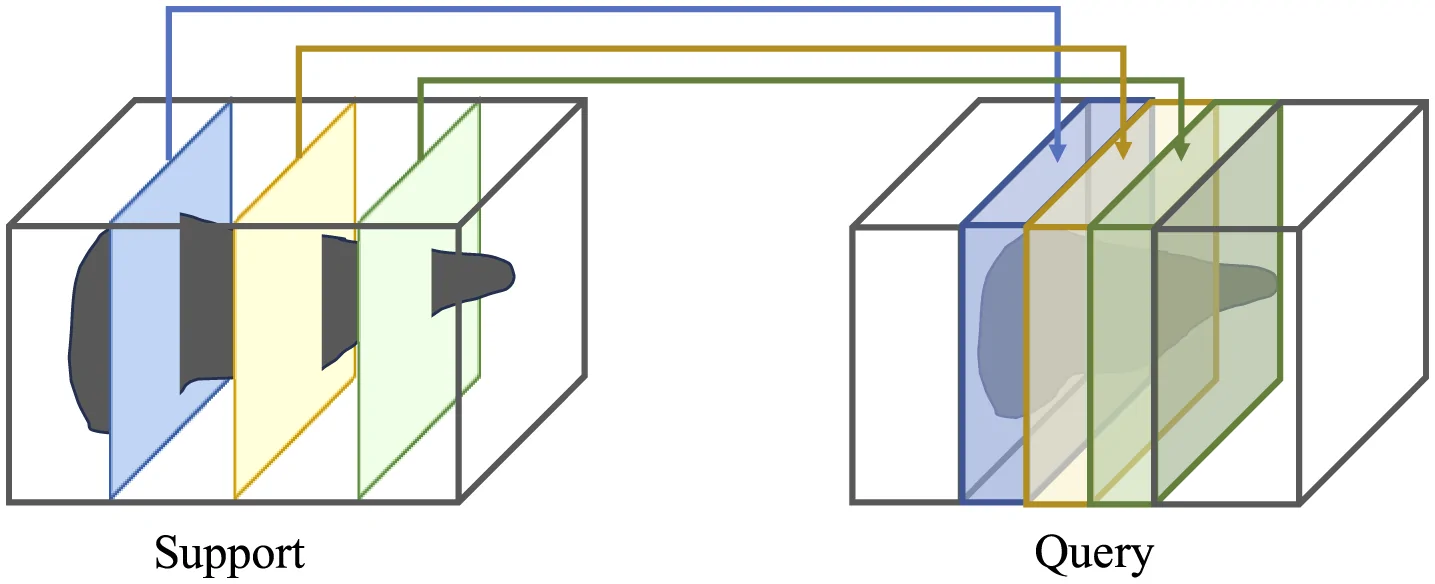
Support-query sampling strategy in EchoSAM2. A clip is split into *K* temporal segments (*K* = 3). The center frame of each segment forms support set S and supervises the corresponding query segment.

To establish a unified and fair annotation budget, we select 10 labeled frames from the training set as the shared labeled pool for all compared methods. For EchoSAM2, at inference time, *K* = 3 frames are randomly sampled from this pool to form the support set S (i.e., a 3-shot setting). For CAMUS, each original video sequence is used directly as the query clip, while for EchoNet-Dynamic, each video is divided into non-overlapping 20-frame clips for inference. As shown in Figure 3, each clip is associated with *K* = 3 support frames. Query segments are processed sequentially, each guided by its matched support frame.

EchoSAM2 uses the SAM2-hiera-small backbone (25) and is trained in two stages.

**Stage I: Registration Network Training**. We train the self-supervised registration model on unlabeled training frames. Random frame pairs from the same video are sampled as moving/fixed images, and the model predicts dense deformation fields optimized by the loss in Section 3.1. The registration network follows the VoxelMorph (32) framework and is trained using the Adam optimizer with a learning rate of 1e-4. Training runs for 1500 epochs with batch size 16. The smoothness weight λ in Equation 2 is set to 0.01 following the recommended setting for MSE-based similarity loss.

**Stage II: Few-shot Video Segmentation**. Stage II is inference-only; all SAM2-hiera-small backbone weights are kept frozen with no gradient updates. The trained registration model aligns support images to query frames. Aligned support image-mask pairs are encoded into support memory, which guides query segmentation through SAM2 memory attention. During inference, frames are processed sequentially using the SAM2 video predictor. Each frame uses both support memory and historical query memory. The memory bank is implemented as an FIFO queue with length *L* = 5.

All experiments use EchoSAM2-hiera-small. The sequential design effectively exploits temporal coherence and produces stable segmentation across cardiac cycles.

### 4.3 Comparison methods

We compare against four groups of baselines. The **first** group contains fully supervised models UNet (43), SwinUNet (44), and SAM-based fully-supervised method MemSAM (42).

The **second** group is UniverSeg (19), a universal few-shot segmentation model trained on diverse medical datasets with support-query style generalization.

The **third** group is the few-shot video baseline MOVE (22), and memory-based video object segmentation methods, STCN (5) and XMem (7). As these are semi-supervised VOS methods requiring a first-frame mask, we provide the registration-warped support mask as their initial annotation.

The **fourth** group includes SAM-family fine-tuning methods: SAMed (10), H-SAM (11), HQ-SAM (12), CAT-SAM (13), and MedicalSAM2 (14). These methods leverage foundation-model priors and can remain competitive under extremely sparse labels.

All methods are evaluated under a unified annotation budget of 10 labeled frames selected from the entire training set (not per patient or per fold). These 10 frames serve different roles depending on the method type. For few-shot methods EchoSAM2, UniverSeg and MOVE, no training on labeled data is required; instead, *K* = 3 frames are randomly sampled from this 10-frame pool at inference time as the support set. For the supervised baselines (UNet, SwinUNet) and SAM-based fine-tuning methods (SAMed, H-SAM, HQ-SAM, CAT-SAM, MedicalSAM2), the same 10 labeled frames are used as the complete training set. This ensures that all methods operate under identical label constraints, enabling a direct comparison of label efficiency. Full-data supervised results (denoted “(ALL)” in Tables 1 and 2) are additionally reported as upper-bound references. None of the compared methods reported few-shot results on CAMUS or EchoNet-Dynamic in their original publications; all of their results in this paper were therefore independently reproduced by us on identical data splits using the official codebases and publicly released model weights.

**Table 1:** CAMUS-Semi 3-way few-shot segmentation performance (LA / LV / MYO and the three-class Mean).

Methods	LA				LV				MYO				Mean			
	Dice ↑	IoU ↑	HD95 ↓	ASSD ↓	Dice ↑	IoU ↑	HD95 ↓	ASSD ↓	Dice ↑	IoU ↑	HD95 ↓	ASSD ↓	Dice ↑	IoU ↑	HD95 ↓	ASSD ↓
UNet (ALL)	89.55	81.40	5.60	2.28	92.40	86.02	4.90	2.00	88.44	79.67	6.81	2.77	90.13	82.36	5.77	2.35
SwinUNet (ALL)	88.26	79.37	5.92	2.52	91.11	83.99	5.19	2.21	87.15	77.64	7.20	3.07	88.84	80.33	6.10	2.60
MemSAM (ALL)	92.73	86.65	3.71	1.52	95.58	91.27	3.25	1.33	91.62	84.92	4.51	1.85	93.31	87.61	3.82	1.57
UNet	73.99	63.96	7.15	4.61	76.84	68.58	6.26	4.04	72.88	62.23	8.70	5.61	74.57	64.92	7.37	4.75
SwinUNet	55.04	48.77	9.18	6.03	57.89	53.39	8.04	5.29	53.93	47.04	11.16	7.34	55.62	49.73	9.46	6.22
MOVE	83.58	75.32	5.01	3.57	86.43	79.94	4.39	3.13	82.47	73.59	6.10	4.34	84.16	76.28	5.17	3.68
STCN	80.02	72.57	5.70	3.79	82.87	77.19	5.00	3.32	78.91	70.84	6.94	4.61	80.60	73.53	5.88	3.91
XMem	81.53	74.08	5.26	3.72	84.38	78.70	4.61	3.26	80.42	72.35	6.40	4.53	82.11	75.04	5.42	3.84
UniverSeg	85.25	76.72	4.48	3.43	88.10	81.34	3.93	3.01	84.14	74.99	5.45	4.18	85.83	77.68	4.62	3.54
SAMed	82.31	73.66	4.76	3.64	85.16	78.28	4.17	3.19	81.20	71.93	5.79	4.43	82.89	74.62	4.91	3.75
H-SAM	86.80	78.51	4.33	2.89	89.65	83.13	3.79	2.53	85.69	76.78	5.26	3.52	87.38	79.47	4.46	2.98
HQ-SAM	82.97	74.30	4.71	3.51	85.82	78.92	4.13	3.08	81.86	72.57	5.73	4.27	83.55	75.26	4.86	3.62
CAT-SAM	81.05	73.40	4.83	3.74	83.90	78.02	4.23	3.28	79.94	71.67	5.88	4.55	81.63	74.36	4.98	3.86
MedicalSAM2	85.71	77.77	4.14	2.44	88.56	82.39	3.63	2.14	84.60	76.04	5.04	2.97	86.29	78.73	4.27	2.52
Ours	**87.46**	**79.31**	**3.77**	**2.21**	**90.31**	**83.93**	**3.31**	**1.94**	**86.35**	**77.58**	**4.59**	**2.69**	**88.04**	**80.27**	**3.89**	**2.28**

Best results among few-shot methods are **bolded**, second-best are underlined.

**Table 2:** EchoNet-Dynamic LV-only (1-way) few-shot segmentation performance.

Methods	mDice ↑	mIoU ↑	HD95 ↓	ASSD ↓
UNet (ALL)	91.36	83.27	4.98	3.01
SwinUNet (ALL)	87.79	80.14	6.61	5.71
MemSAM (ALL)	92.78	85.89	4.57	2.71
UNet	76.21	65.32	8.47	5.92
SwinUNet	57.46	52.71	9.84	8.16
MOVE	83.89	75.42	5.54	4.81
STCN	79.82	71.67	6.03	5.72
XMem	81.47	72.13	5.71	5.19
UniverSeg	84.26	76.13	4.82	4.07
SAMed	81.73	73.95	6.01	5.26
H-SAM	86.04	77.41	4.91	3.21
HQ-SAM	82.61	74.13	5.66	4.37
CAT-SAM	80.72	72.39	6.24	5.44
MedicalSAM2	84.09	75.87	4.82	3.57
Ours	**87.33**	**80.41**	**4.56**	**3.17**

All metrics are computed on the single LV-endocardium class. Best results among few-shot methods are **bolded**, second-best are underlined.

## 5 Results

### 5.1 Quantitative comparison with state-of-the-art methods

Tables 1 and 2 report quantitative comparisons on CAMUS-Semi (3-way) and EchoNet-Dynamic (LV-only), respectively. EchoSAM2 achieves the best overall performance under the few-shot setting, and the improvement is consistent across both overlap-based and boundary-based metrics. This is important because improvements in Dice/IoU alone may still hide contour instability, whereas simultaneous gains in HD95/ASSD indicate that the predicted boundaries are also more anatomically plausible.

On CAMUS, EchoSAM2 reaches 88.04% mDice and 80.27% mIoU, exceeding H-SAM by 0.66% and 0.80%, respectively. More importantly, boundary quality is also best, with HD95 = 3.89 and ASSD = 2.28. Compared with MedicalSAM2, which is competitive in boundary metrics, EchoSAM2 still achieves lower HD95 and ASSD while keeping higher overlap scores, indicating a better balance between region coverage and contour precision. Table 1 further breaks performance down by structure. Across methods, the large LV cavity is the easiest to segment, LA is intermediate, and the thin, low-contrast MYO is the hardest. EchoSAM2 ranks first on every structure (87.46%, 90.31%, and 86.35% Dice on LA, LV, and MYO), with the largest gain on the challenging MYO, indicating that its advantage is spread across all classes rather than driven by a single easy one.

On EchoNet-Dynamic, EchoSAM2 attains 87.33% mDice and 80.41% mIoU, outperforming H-SAM by 1.29% and 3.00%. It also obtains the best HD95 (4.56) and a highly competitive ASSD (3.17). Notably, EchoNet-Dynamic provides sparse supervision (ED/ES only), so robust performance in this setting suggests that our sequence-wise memory design can transfer effectively even when direct temporal labels are limited.

Compared with supervised baselines trained on 10 slices, EchoSAM2 shows large gains. On CAMUS, mDice increases from 74.57% (UNet) and 55.62% (SwinUNet) to 88.04%; on EchoNet-Dynamic, similar trends are observed. Although full-supervision UNet remains stronger, EchoSAM2 narrows the gap substantially despite using only 10 labels, highlighting its label-efficiency advantage.

Compared with UniverSeg, EchoSAM2 is consistently stronger on both overlap and boundary metrics across the two datasets. This result indicates that large-scale generic pretraining is valuable but still insufficient without task-specific temporal modeling and support-query alignment tailored to echocardiography.

Compared with MOVE, EchoSAM2 delivers clear gains, especially in HD95 and ASSD. This suggests that our method improves not only temporal smoothness but also local contour fidelity, which is critical for quantitative cardiac measurements. Compared with the memory-based VOS methods STCN and XMem, EchoSAM2 exceeds the stronger XMem by 5.93% mDice and lowers ASSD from 3.84 to 2.28 on CAMUS, with similar margins on EchoNet-Dynamic. Their natural-video priors and reliance on a propagated first-frame mask without support conditioning even leave them behind MOVE and UniverSeg.

Among SAM-based adaptation baselines, EchoSAM2 offers the strongest overall balance of overlap accuracy and boundary precision, demonstrating that combining domain-adapted memory propagation, adaptive initialization, and registration is more effective than backbone adaptation alone.

### 5.2 Qualitative analysis

**Figure 4:**
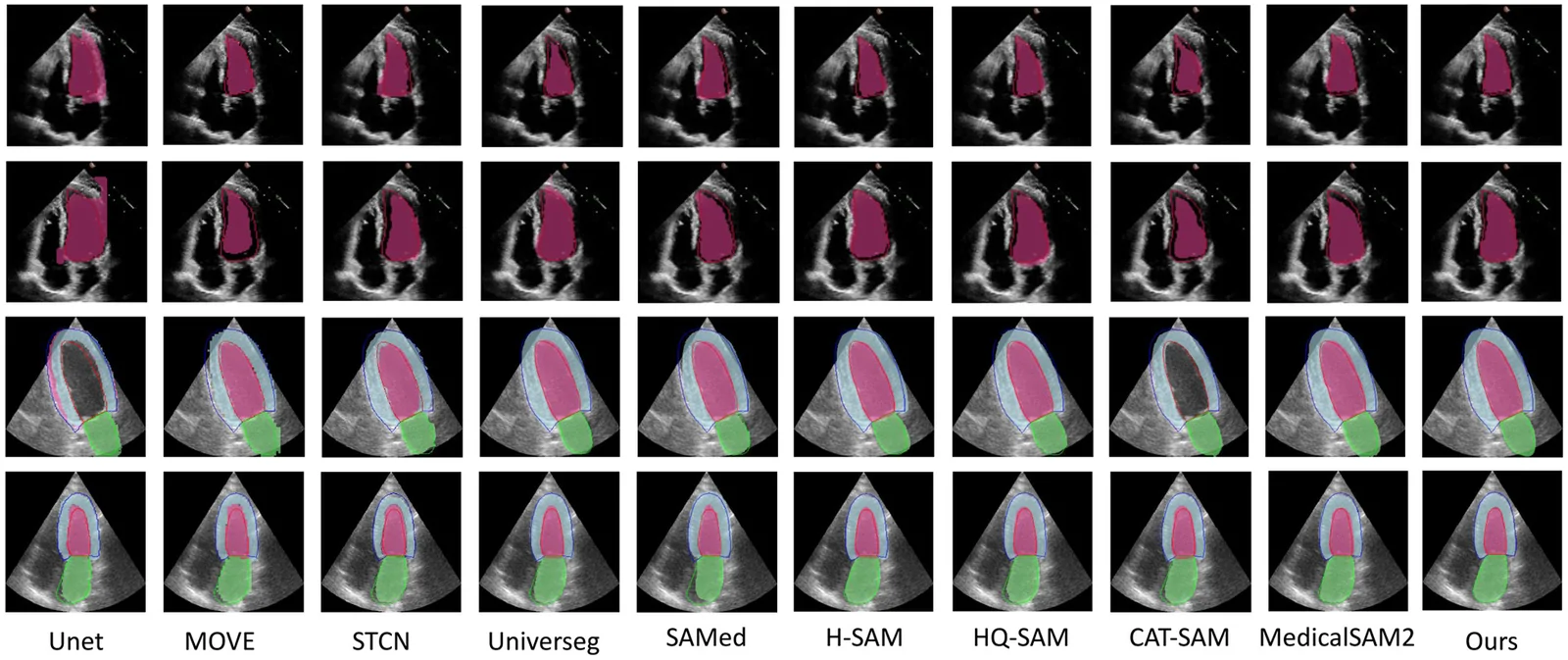
Qualitative segmentation results. **Top two rows**: EchoNet-Dynamic LV-only cases; **bottom two rows**: CAMUS three-class cases. Ground-truth contours are drawn as solid curves (green: LA, red: LV, blue: MYO), and the corresponding predictions are shown as regions in a lighter shade of the same color.

Representative visualizations are shown in Figure 4. Fully supervised models such as UNet can recover coarse anatomy but often produce imprecise and blurry boundaries in difficult regions. MOVE benefits from temporal context, yet still struggles with fine contour localization and may show local leakage near low-contrast borders.

SAM-based baselines (SAMed, HQ-SAM, CAT-SAM, MedicalSAM2) improve localization after adaptation but frequently generate coarse masks and miss subtle structures. H-SAM performs better than most SAM variants, though boundary stability remains limited in complex cases, especially when local texture changes rapidly across adjacent frames.

In contrast, EchoSAM2 yields sharper and more coherent predictions across the sequence. By combining support-conditioned memory, adaptive start-frame selection, and registration-guided alignment, it better preserves thin anatomical details and maintains contour continuity over time. This visual trend is consistent with the improvements observed in HD95 and ASSD, supporting that our gains are not only numerical but also anatomically meaningful.

### 5.3 Temporal evaluation on consecutive frames

**Figure 5:**
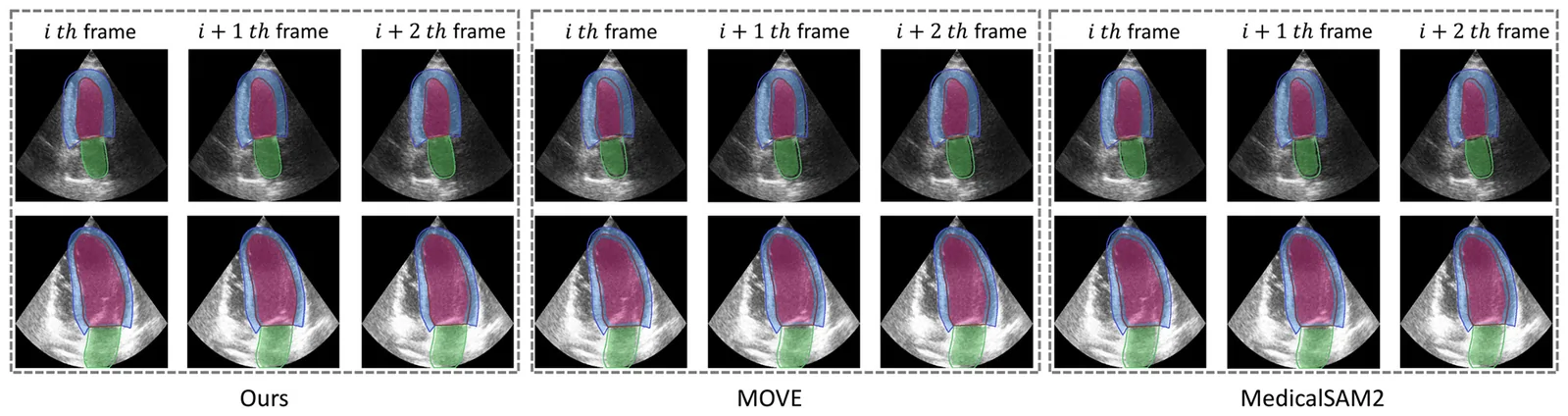
Consecutive-frame segmentation on CAMUS. EchoSAM2 maintains temporally coherent contours across adjacent frames with little flickering.

The comparison above is computed on ED/ES frames, consistent with the CAMUS-Semi and EchoNet-Dynamic settings. To directly assess segmentation quality and temporal coherence over the full cardiac cycle, we additionally exploit the dense per-frame annotations available in CAMUS and evaluate on all consecutive annotated frames of each test video. Table 3 reports the per-frame Dice, IoU, HD95, and ASSD averaged over the whole sequence. EchoSAM2 attains the best per-frame accuracy across the cycle, indicating that its predictions remain stable between consecutive frames rather than only at the two key frames. Figure 5 shows consecutive-frame predictions for a representative sequence: EchoSAM2 produces smooth, anatomically coherent contours with little frame-to-frame flickering, whereas frame-independent baselines exhibit visible jitter near the boundary. These results provide direct, ground-truth-based evidence for the temporal-coherence claims over consecutive frames.

**Table 3:** Temporal evaluation on CAMUS using the dense per-frame annotations across the cardiac cycle, reported per structure (LA / LV / MYO) and as the three-class mean.

Methods	LA				LV				MYO				Mean			
	Dice ↑	IoU ↑	HD95 ↓	ASSD ↓	Dice ↑	IoU ↑	HD95 ↓	ASSD ↓	Dice ↑	IoU ↑	HD95 ↓	ASSD ↓	Dice ↑	IoU ↑	HD95 ↓	ASSD ↓
MOVE	82.31	74.18	5.43	3.81	85.17	77.53	4.72	3.28	81.24	72.69	6.39	4.62	82.91	74.80	5.51	3.90
XMem	80.42	73.04	5.71	4.01	83.27	76.28	5.02	3.48	79.31	71.48	6.67	4.81	81.00	73.60	5.80	4.10
H-SAM	85.03	77.31	4.73	3.11	87.92	80.57	4.01	2.58	83.91	75.82	5.66	3.91	85.62	77.90	4.80	3.20
MedicalSAM2	84.12	76.28	4.52	2.81	86.98	79.61	3.81	2.28	83.04	74.81	5.47	3.61	84.71	76.90	4.60	2.90
EchoSAM2 (Ours)	**86.51**	**78.71**	**4.02**	**2.43**	**89.37**	**82.04**	**3.31**	**1.92**	**85.42**	**77.15**	**4.97**	**3.15**	**87.10**	**79.30**	**4.10**	**2.50**

Metrics are averaged over all consecutive annotated frames. Best results among few-shot methods are in **bold**.

### 5.4 Clinical correlation analysis

To verify that the predicted masks support reliable functional measurement, we perform a clinical correlation analysis on EchoNet-Dynamic, which provides a reference left-ventricular ejection fraction (LVEF) for each study. From the predicted ED and ES LV masks we estimate the LV volumes using the area–length method and compute LVEF as EF = (*V*_*ED*_−*V*_*ES*_)/*V*_*ED*_×100%; since EF is a volume ratio, the pixel-to-physical scaling cancels and no explicit calibration is required. The estimated EF is then compared with the reference EF using the Pearson correlation coefficient (*r*), the mean absolute EF error (MAE, in EF points), and the Bland–Altman bias with 95% limits of agreement. As reported in Table 4, EchoSAM2 attains a Pearson correlation of *r* = 0.87, a mean absolute EF error of 6.1 EF points, and a small Bland–Altman bias of −0.8% (limits of agreement ±11%), outperforming the strongest baselines H-SAM (*r* = 0.81, MAE = 7.4) and MedicalSAM2 (*r* = 0.83, MAE = 7.0). The high correlation together with the small bias indicates that the segmentations produced by EchoSAM2 are accurate enough to support reliable cardiac functional quantification, substantiating the clinical-relevance claim.

**Table 4:** Clinical correlation analysis on EchoNet-Dynamic.

Method	Pearson *r* ↑	EF MAE (pts) ↓	Bias (%)
H-SAM	0.81	7.4	−1.6
MedicalSAM2	0.83	7.0	−1.2
EchoSAM2 (Ours)	**0.87**	**6.1**	**−0.8**

LVEF is estimated from the predicted ED/ES LV masks (area–length method) and compared with the reference EF. Pearson *r*, mean absolute EF error (MAE, EF points), and Bland–Altman bias are reported. Best results in **bold**.

### 5.5 Ablation studies

#### 5.5.1 Effect of inference start position

Table 5 compares fixed starting positions within each temporal segment—Top (first frame of the segment), Middle (central frame), Bottom (last frame), Random (randomly chosen frame), and ASFS. Among fixed choices, Middle generally performs best, likely because central frames often contain relatively complete and stable anatomy. Nevertheless, ASFS consistently outperforms all fixed/random strategies on both datasets. Relative to the best fixed setting, ASFS increases mDice and improves boundary metrics, confirming that adaptive start-frame selection is beneficial for sequence inference. These observations indicate that initialization quality has a nontrivial impact: poor starts can introduce early errors that propagate through memory, while ASFS mitigates this effect by selecting more informative starting frames.

**Table 5:** Effect of different inference start position strategies on CAMUS and EchoNet-Dynamic.

Start Position	CAMUS				EchoNet-Dynamic			
	mDice ↑	mIoU ↑	HD95 ↓	ASSD ↓	mDice ↑	mIoU ↑	HD95 ↓	ASSD ↓
Top	86.21	78.34	4.62	2.91	85.47	77.68	5.18	3.62
Middle	87.42	79.51	4.18	2.53	85.23	78.11	4.73	3.29
Bottom	85.76	77.89	4.85	3.05	84.93	77.21	5.36	3.78
Random	86.03	78.12	4.71	2.97	85.46	78.73	4.79	3.31
ASFS (Ours)	**88.04**	**80.27**	**3.89**	**2.28**	**87.33**	**80.41**	**4.56**	**3.17**

“Top”, “Middle”, “Bottom”, and “Random” denote fixed starting positions at the first, central, last frame, and randomly chosen frame of each temporal segment (see Figure 3), respectively. The best results are bolded, and the second results are underlined.

#### 5.5.2 Robustness of ASFS to support-mask quality

We further examine whether a low-quality mask biases start-frame selection, because the ROI Ω_*j*_ is defined by the warped support mask. We degrade the warped support mask by morphological erosion/dilation (±5 px) and random boundary jitter, and re-evaluate segmentation accuracy. As shown in Table 6, ASFS degrades only marginally and still surpasses the best fixed-start strategy (Middle) under all degradation levels, because the ROI only needs to coarsely localize the target structure to suppress background interference in the MSE and does not require a pixel-accurate mask.

**Table 6:** Robustness of ASFS to support-mask quality on CAMUS (mDice, %).

Support-mask quality	Fixed-middle	ASFS (Ours)
Clean	87.42	**88.04**
Erode 5 px	87.04	**87.71**
Dilate 5 px	86.53	**87.36**
Boundary noise	86.26	**87.08**

The warped support mask defining the ROI is degraded by erosion/dilation and boundary jitter. ASFS outperforms the best fixed-start strategy under all levels. The best results are bolded.

#### 5.5.3 Effect of registration

Because support memory quality depends on support-query alignment, we evaluate three settings: no registration, affine registration (ANTs), and our lightweight registration network. As shown in Table 7, removing registration degrades segmentation quality on both datasets, indicating that misalignment weakens memory guidance. This setting is fastest (5.29 s/video), but accuracy drops.

**Table 7:** Effect of different registration strategies on segmentation performance and inference efficiency.

Registration	CAMUS				EchoNet-Dynamic				Inference time
	mDice ↑	mIoU ↑	HD95 ↓	ASSD ↓	mDice ↑	mIoU ↑	HD95 ↓	ASSD ↓	T/video (s) ↓
w/o Reg	86.12	78.05	4.72	2.94	85.01	77.36	5.31	3.61	**5.29**
w/ Affine Reg	87.95	80.11	3.95	2.35	**87.42**	**80.53**	4.60	3.20	12.73
w/Reg network (Ours)	**88.04**	**80.27**	**3.89**	**2.28**	87.33	80.41	**4.56**	**3.17**	5.37

We compare no registration, conventional affine registration, and the proposed lightweight registration network. The best results are bolded, and the second results are underlined.

Affine registration significantly boosts overlap and boundary quality, showing that geometric alignment is beneficial; however, runtime increases substantially (12.73 s/video), which limits practical use in efficiency-sensitive settings. Our registration network offers the best accuracy-efficiency balance: it attains top CAMUS scores and best HD95/ASSD on EchoNet-Dynamic while adding only minimal overhead (5.37 s/video). Overall, the results suggest that learned non-rigid alignment is effective and lightweight enough for practical deployment.

#### 5.5.4 Effect of memory queue length

Table 8 analyzes the effect of the memory queue length *L* on CAMUS, reported per structure. The case *L* = 0 corresponds to frame-wise inference, where the memory bank stores only the support memory $M_{s}$, with a mean Dice of 83.80%. Introducing memory from historical query frames (*L*>0) leads to clear gains, and the mean Dice peaks at *L* = 5 (our default) with 88.04%, a 4.24% improvement over the frame-wise inference. A per-structure analysis reveals a structure-dependent trade-off. For the large and stable LV, accuracy keeps improving as *L* grows and is highest at *L* = 7 (90.55%): a longer queue provides more redundant temporal context, improving temporal consistency, reducing flickering, and making predictions more robust to single-frame errors. In contrast, for the thin and rapidly deforming MYO boundary, an overly long queue is detrimental where MYO Dice drops from 86.35% at *L* = 5 to 85.90% at *L* = 7, because stale memories from earlier cardiac phases no longer match the current fine boundary and dilute the recency signal. Since the mean is dominated by this degradation of small structures, the overall optimum is reached at the moderate length *L* = 5, which we adopt as the default.

**Table 8:** Effect of different memory queue lengths *L* on the CAMUS dataset (DSC %).

Memory Length *L*	CAMUS (DSC % ↑)			
	LA	LV	MYO	Mean
0 (Frame-wise)	83.40	86.00	82.00	83.80
1	85.20	88.00	84.10	85.77
3	86.50	89.40	85.70	87.20
5 (Ours)	**87.46**	90.31	**86.35**	**88.04**
7	87.30	**90.55**	85.90	87.92

*L* = 0 corresponds to frame-wise inference where the memory bank stores only the support memory $M_{s}$. Best results in **bold**, second-best are underlined.

## 6 Discussion and conclusion

We introduced EchoSAM2, a few-shot echocardiography segmentation framework that integrates sequence-wise memory propagation, adaptive inference initialization, and efficient support-query registration. Experiments show that this combination substantially improves performance under sparse supervision and provides a practical route for low-label clinical deployment.

A central finding is the value of sequence-aware modeling. Compared with frame-independent inference, our approach improves both overlap and boundary quality by leveraging temporal continuity. At the same time, sequence propagation depends on reliable initialization; ASFS addresses this by selecting better starting frames and reducing the accumulation of early prediction errors.

We also confirm the importance of alignment. Traditional affine registration can improve results but is computationally expensive and limited for non-rigid motion. Our lightweight learned registration achieves a stronger accuracy-efficiency trade-off, preserving most of the computational advantages of the no-registration setting while delivering better segmentation quality.

The results further indicate that SAM-style pretraining alone is insufficient for echocardiography without domain-specific adaptation. Temporal memory design, support conditioning, and alignment-aware initialization are all necessary to transfer foundation-model priors to noisy and dynamic ultrasound data.

This study still has limitations: the current setting focuses on single-modality ultrasound, does not explicitly model multi-view or multi-modal information, and may require further acceleration for strict real-time use. Future work will extend EchoSAM2 to multi-view/multi-modal data, explore vision-language integration for clinical prior injection, and improve robustness under severe anatomical variation and low-quality acquisition conditions. Overall, EchoSAM2 offers an effective and practical solution for few-shot echocardiography segmentation with clear promise for clinical deployment.

## Data Availability

The original contributions presented in the study are included in the article/supplementary material, further inquiries can be directed to the corresponding author.
